# MiRNAs in Poultry Health and Production: Progress and Challenges

**DOI:** 10.3390/ani15223230

**Published:** 2025-11-07

**Authors:** Thanh Van Nguyen, Tan Hy Tat, Duy Ngoc Do

**Affiliations:** 1 Faculty of Veterinary Medicine, Viet Nam National University of Agriculture, Hanoi 100000, Vietnam; thanhnv1102@gmail.com; 2 Faculty of Animal Science and Veterinary Medicine, Ho Chi Minh City University of Agriculture and Forestry (HCMUAF), Ho Chi Minh City 700000, Vietnam; tanhytat@gmail.com; 3 Institute of Research and Development, Duy Tan University, Da Nang 550000, Vietnam; 4 School of Medicine and Pharmacy, Duy Tan University, Da Nang 550000, Vietnam; 5 Department of Animal Science and Aquaculture, Dalhousie University, Truro, NS B2N 5E3, Canada

**Keywords:** MiRNAs, regulatory network, poultry

## Abstract

MicroRNAs (miRNAs) are small, non-coding RNA molecules that play crucial roles in the regulation of gene expression. In poultry, miRNAs have emerged as significant regulators of various biological processes, such as growth, development, immune response, and disease resistance in humans, plants, and animals. Recent studies have identified numerous miRNAs in different poultry species (chickens, geese, turkeys, quails, and ducks) and have elucidated their roles in various economically important traits in these species. This review aims to provide a comprehensive overview of the progress made in understanding the functions and mechanisms of miRNAs in poultry as well as the challenges faced in this field. Firstly, an overview of progress in the identification and validation of miRNAs in each species is provided. Then, specific roles of miRNAs in different classes of phenotypic traits are presented. Finally, the challenges and impacts of key technologies and methods, including artificial intelligence, genome editing, and single-cell sequencing, are discussed. From state-of-the-art research on miRNA functions across different trait classes to up-to-date technology trends, this review provides a comprehensive picture of miRNAs in poultry, which might open up new research and application possibilities.

## Simple Summary

MicroRNAs (miRNAs) are small molecules that control gene expression, and they are proving to be important players in poultry biology. We summarize updated findings on miRNA research in chickens, ducks, turkeys, and geese, focusing on how they influence traits like growth, immunity, and disease resistance. We also explore how new tools and methods—such as machine learning and genome editing—are changing the way we study these molecules. By combining past research with emerging technologies, this review presents a full picture of miRNAs’ role in poultry and highlights exciting future possibilities.

## 1. Introduction

Poultry production is one of the most important types of livestock production in the world and is vital for the global agricultural sector. Among poultry species, chickens are the most widely produced species due to their high efficiency in converting feed into protein. With operations ranging from small backyard flocks to large-scale commercial operations, poultry farming is vital in satisfying the growing demand for animal protein, especially in developing nations where population increase and urbanization are driving consumption [1]. Genetics, nutrition, disease management, and production technologies have transformed poultry production by increasing productivity, improving animal welfare, and reducing environmental impacts [2]. However, the industry also faces challenges, such as disease outbreaks, antibiotic resistance, and sustainability concerns, which necessitate continuous research and adaptation to ensure its long-term viability.

First identified in 1993, microRNAs (miRNAs) have been added to the new layer in regulatory networks in almost all biological processes. They are small, non-coding RNA molecules, typically 20–24 nucleotides in length, that play a critical role in post-transcriptional gene regulation [3]. They function by binding to complementary sequences on target messenger RNAs (mRNAs), leading to mRNA degradation or translational repression, thereby fine-tuning gene expression [4]. Since their discovery, miRNAs have been recognized as key regulators of diverse biological processes, including development, cell differentiation, apoptosis, and immune response [5]. MicroRNAs are highly conserved across species, underscoring their fundamental importance in cellular function. They are transcribed from genomic DNA and undergo a series of processing steps involving the Drosha and Dicer enzymes to produce mature miRNA molecules [6]. Dysregulation of miRNAs has been implicated in various diseases, including cancer, cardiovascular disorders, and neurodegenerative conditions, making them potential biomarkers for diagnosis and therapeutic targets [7]. The study of miRNAs continues to expand our understanding of gene regulation and its implications for health and disease in all living animals. Understanding the regulatory functions of miRNAs in poultry is crucial for the development of novel strategies for improving poultry health, productivity, and disease resistance.

The existing review papers provide valuable insights into miRNAs’ roles in several aspects related to skeletal muscle development, lipid metabolism, viral diseases, and host–pathogen interaction in chicken [8,9,10,11,12]. However, these reviews only focus on a few traits, are not specific to miRNAs, or are not up to date. In addition, these reviews are only on chicken, without information on other poultry species. A new review is warranted to integrate recent findings, address gaps in mechanistic understanding, and focus on practical applications for the poultry industry. Therefore, this review provides an overview of progress in the identification and validation of miRNAs in each species. It will then present the specific roles of miRNAs in different classes of phenotypic traits and discuss the challenges and impacts of key technologies and methods, such as artificial intelligence (AI) and genome editing.

## 2. Poultry Genomes and MicroRNAs Research

Owing to the advances in sequencing, the genomes of major poultry species have been completed (Table 1). They reveal significant diversity in size, gene count, and microRNA (miRNA) profiles, reflecting their evolutionary adaptations and selection purposes. Being the most popular poultry species, the genome of the chicken (*Gallus gallus*) is the most complete one. The first complete genome of chickens was released in 2004 [13], and the chicken genome has been updated several times since then. Currently, chicken has a genome size of 1.1 Gb and 18,023 genes and 474 miRNAs. Turkeys (*Meleagris gallopavo*) and ducks (*Anas platyrhynchos*) have slightly larger genomes at 1.1 Gb and 1.3 Gb, respectively, with turkeys having about 17,974 genes and 353 miRNAs and ducks having around 17,676 genes and 300 miRNAs. Geese (*Anser anser*) and quails (*Coturnix japonica*) also show considerable genomic complexity, with geese having a genome size of 1.3 Gb, with about 19,000 genes and 250 miRNAs, and quails having a genome size of 1.24 Gb, with around 17,000 genes and 200 miRNAs. Other species like guinea fowl (*Numida meleagris*), ostriches (Struthio camelus), and emus (*Dromaius novaehollandiae*) exhibit varying genome sizes and gene counts, with limited data on their miRNA profiles.

**Figure 1:**
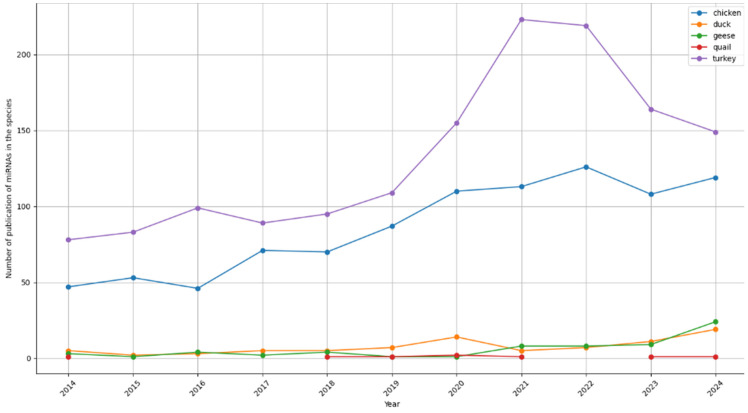
Overview of research trends in miRNAs in major poultry species published in the National Center for Biotechnology Information (NCBI) up to 2024. The y-axis shows the number of publications by year based on searches in the PubMed database (https://www.ncbi.nlm.nih.gov/, accessed on 20 January 2025) using the keywords “species” and “miRNAs” or “microRNA”.

Several reviews have been conducted to summarize information on the roles of miRNAs in different traits of poultry species [10,14,15,16]. The number of studies in major poultry species increased quickly from 2014 to 2024, according to the PubMed database (https://www.ncbi.nlm.nih.gov/, accessed on 20 January 2025) (Figure 1). Some other recent reviews focus only on muscle development, diseases, or heat stress in chicken, which might not cover all the aspects of miRNA’s regulatory network in poultry. This review organizes miRNA information based on trait ontology, which makes it easy to obtain up-to-date knowledge on the roles of miRNAs. In addition, given the fact that AI has been a popular application in several aspects of research, the potential applications of AI will also be discussed.

## 3. Potential Functions of microRNAs in Poultry

### 3.1. MicroRNAs in Chicken

To meet the rising demand for white meat, the poultry sector primarily focuses on improving feed efficiency and breast yield. Beyond targeted success in genetic/genomic selection for leaner and smaller chickens, these approaches have greatly saved resources and feeding costs while raising production and lowering greenhouse gas emissions. More comprehensive studies on miRNAs have been conducted in chickens, including the validations of miRNAs from different tissues and organs. While expression profiles are well established in all major chicken tissues, the functional studies of miRNAs remain ongoing.

#### 3.1.1. MicroRNAs Related to Production Traits in Chicken

##### MicroRNAs Involved in Muscle Growth and Meat Quality in Chickens

Muscle growth and breast muscle development are critical traits in poultry production, as they directly influence meat yield and quality [17]. In chickens, several miRNAs have been identified as key regulators of muscle growth, affecting processes such as myogenesis (muscle formation), satellite cell proliferation, and muscle fiber development (Table 2). Among these miRNAs, miR-1 and miR-206 are abundant in the muscle tissue [18,19] and are often referred to as myomiRs due to their essential roles in muscle development. MicroRNA-1 promotes muscle differentiation by targeting genes that inhibit myogenesis [20]. For example, miR-1 targets histone deacetylase 4 (HDAC4) [9,21,22,23], a transcriptional repressor of muscle differentiation. By downregulating HDAC4, miR-1 enhances the expression of muscle-specific genes, such as myogenin and MyoD, which are critical for muscle development [22]. Moreover, miR-1 is highly expressed during embryonic muscle development and is essential for the formation of skeletal muscle [24].

Meanwhile, miR-206 has been reported to promote muscle differentiation and regeneration, as it targets Paired box protein Pax-7 (Pax7), a gene involved in maintaining satellite cell population (muscle stem cells) [25]. By downregulating Pax7, miR-206 facilitates the transition of satellite cells into differentiated muscle cells [25]. Upregulated miR-206 during muscle growth is associated with the development of fast-twitch muscle fibers, which are important for meat production. Some other important miRNAs for regulating muscle development and meat quality include miR-133, miR-27a, miR-499, miR-208, miR-29, and miR-486. miR-133 is a muscle-specific miRNA that plays a dual role in muscle growth. It promotes muscle proliferation while inhibiting differentiation during early stages of myogenesis. MicroRNA-133 targets serum response factor (SRF), a transcription factor that regulates muscle-specific genes. By downregulating SRF, miR-133 promotes the proliferation of myoblasts, ensuring an adequate pool of muscle precursor cells [26,27]. MicroRNA-27a targets Peroxisome Proliferator-Activated Receptor Gamma (PPARγ), a key regulator of adipogenesis (fat cell formation), and works as a regulator for the balance between muscle and fat deposition, which is important for meat quality [28]. MicroRNA-140-5p and miR-18b-3p influence intramuscular fat deposition in chickens, thereby affecting meat quality [29,30]. Importantly, these miRNAs operate within distinct regulatory networks to control muscle development. For instance, miR-1 and miR-133 function as a dynamic duo, working together but with complementary roles that maintain a delicate balance between myoblast proliferation and differentiation. As another example, miR-206 interacts with the IGF-1 signaling pathway by targeting IGF1R or PI3K/AKT pathway components, influencing muscle hypertrophy. Simultaneously, other miRNAs like miR-29 may modulate collagen synthesis in connective tissues, affecting muscle quality.

Chickens possess both fast-twitch (glycolytic) and slow-twitch (oxidative) muscle fibers, with fast-twitch fibers being more abundant in breast muscle. miR-499 targets SRY-Box Transcription Factor 6 (SOX6), a transcriptional repressor of slow-twitch muscle fiber genes [31]. By downregulating SOX6, miR-499 promotes the formation of slow-twitch muscle fibers, which are more oxidative and fatigue-resistant [32]. miR-499 is associated with the development of slow-twitch fibers in leg muscles, which are important for sustained activity [33]. miR-208 is involved in muscle hypertrophy (an increase in muscle size) and is expressed in response to muscle stress or exercise. MicroRNA-208 targets myostatin (MSTN), a negative regulator of muscle growth [34]. By downregulating myostatin, miR-208 promotes muscle hypertrophy and increases muscle mass [35].

##### MicroRNAs in Egg Production-Related Traits

Egg production and quality are crucial traits in poultry, influenced by both genetic and environmental factors as well as their interactions. Key traits include the number of eggs produced, egg weight, shell thickness, shell strength, and albumen quality. Studies have identified specific miRNAs that are differentially expressed in high-rate versus low-rate egg-producing chickens. For example, miRNAs in the pituitary and hypothalamus have been shown to regulate pathways related to ovarian steroidogenesis and hormone biosynthesis, which are essential for egg production. By targeting specific mRNAs, miRNAs can either enhance or suppress the production of proteins involved in these pathways, thereby affecting the egg-laying performance of hens.

Comparative studies of chickens with high and low rates of egg production have identified 17 significantly differentially expressed miRNAs. Known miRNAs, such as miR-34b, miR-34c, miR-216b, and miR-200a-3p, are involved in processes like proliferation, cell cycle, apoptosis, and reproductive regulation [36]. MicroRNA–mRNA pairs miR-200a-3p-SFRP4, miR-101-3p-BMP5, miR-32-5p-FZD4, and miR-458b-5p-CTNNB1 are potentially associated with ovarian development [37]. MicroRNA-34 has an important impact on the reproductive process of hens [38], as it might regulate processes such as proliferation, cell cycle, apoptosis, and metastasis, and is expressed differentially in the ovaries of chickens with high rates of egg production [39]. These processes play an important role in ovary development and reproductive regulation in chickens [39]. A coordinated network of miRNAs—including miR-26a, miR-148a, and miR-10a—plays a pivotal role in regulating ovarian function and optimizing egg production [40]. These miRNAs target key genes involved in follicular development (FSHR and TGFB1), steroidogenesis (CYP19A1 and STAR), and hormonal signaling (ESR1), thereby influencing estrogen synthesis and oviduct integrity. Their activity is modulated by transcription factors like FOXL2, which forms feedback loops to sustain follicular growth [41]. Additionally, variants in miRNAs have also been reported to be significantly associated with egg production. Polymorphisms in pri-miR-26a-5p (the primary transcript of miR-26a-5p) are significantly associated with important egg production traits in chickens, such as age at first egg and total number of eggs at 32 weeks [42], while SNP rs737028527 (G > A) affects miR-1b-3p biogenesis and its effects on chicken egg-laying traits [43].

##### MicroRNA in Chicken Feed Efficiency

Although it is one of the most important traits for the poultry industry, very few studies have been devoted to understanding the mechanism of feed efficiency. In a genomic study, miR-15a was linked to the control of the feed conversion ratio in layer chickens [44], influencing genes in the insulin signaling pathway, which is known to regulate appetite and feed intake. Luo et al. also reported that an SNP in miR-1596 is important for residual feed intake, an important indicator of feed efficiency in chickens [45]. Two miRNAs, miR-1730 and miR-1744, are linked to the feed conversion ratio (FCR), a trait in broilers [46]. The DEG analyses by [47] reported that miR-30a-5p, miR-181a-5p, and miR-206 were significantly upregulated in the pectoral muscle of broiler chickens fed a phytobiotic-supplemented diet, which improved FCR, a key measure of feed efficiency. Conversely, miR-99a-5p, miR-133a-5p, miR-142-5p, and miR-222 were downregulated under the same conditions, suggesting their involvement in the regulation of feed efficiency traits [47]. Another crucial process linked to feed efficiency is fat deposition. Tian et al. showed that miR-34a-5p targeted Acyl-CoA Synthetase Long Chain Family Member 1 (ACSL1) protein expression to increase hepatic triglycerides and total cholesterol levels in laying hens [48]. Since feed accounts for 70% of total production cost in the poultry sector, it is crucial for functional studies of miRNAs to be continuously developed to increase feed efficiency in chickens. Selection of divergent feed-efficient animals might be feasible using several miRNAs (miR-1730, miR-1759, and miR-1744).

#### 3.1.2. MicroRNAs in Chicken Immune Functions and Diseases

MicroRNAs play a critical role in regulating chicken health and disease resistance by controlling key processes such as immune response, inflammation, apoptosis, and immune cell differentiation. Understanding the roles of these miRNAs provides valuable insights into the molecular mechanisms underlying disease resistance and offers potential applications for improving chicken health and disease resistance in poultry breeding programs.

For instance, miR-155 is a well-known immune-related miRNA that enhances the immune response by targeting the Suppressor of Cytokine Signaling 1 (SOCS1) gene [49,50]. SOCS1 is a negative regulator of the innate immune response, and by downregulating SOCS1, miR-155 promotes the expression of pro-inflammatory cytokines and enhances immune cell differentiation. MicroRNA-155 is highly expressed in immune cells and is associated with improved resistance to viral and bacterial infections [49,51]. In chickens, miR-155 is important for stress-induced immunosuppression mechanisms, affecting immune response to different vaccines in chickens [50,52]. It is also important for the response to reticuloendotheliosis virus infection in chicken embryo fibroblasts [53]. Numerous studies have identified specific miRNAs as key regulators and potential biomarkers in major poultry diseases, including Marek’s disease, avian leukosis, infectious bursal disease, avian influenza, and chronic respiratory diseases [11,12,54,55]. The potential regulatory mechanism and expressed tissues of miRNAs for each type of infection and related disease are shown in Table 3.

##### Regulation of Bacterial Infections

**Figure 2:**
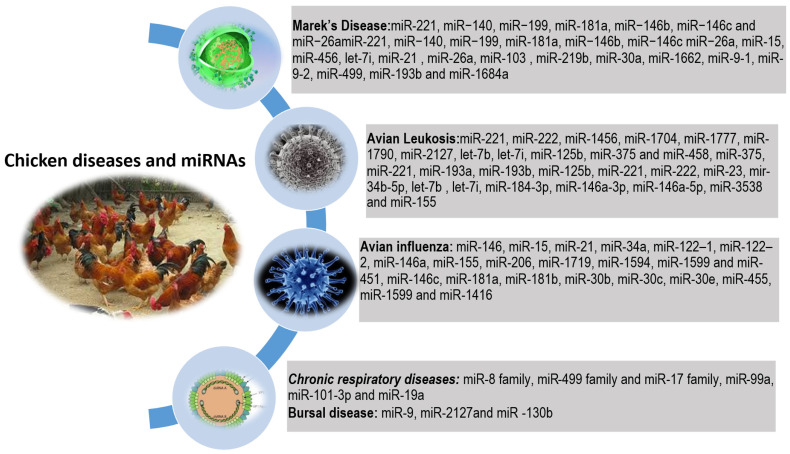
MiRNAs in important chicken diseases.

Bacterial infections are a significant threat to chicken health, and miRNAs have been shown to play a role in the immune response to bacterial pathogens (Figure 2). For example, miR-199 is involved in the immune response to bacterial infections by targeting the nuclear factor kappa B (NF-κB) pathway. NF-κB is a key regulator of immune response and inflammation, and by modulating this pathway, miR-21 enhances the immune response to bacterial infections [56]. In chickens, miR-199 is upregulated during bacterial infections and is associated with improved disease resistance. Another miRNA, miR-33, is involved in regulating cholesterol homeostasis [57], which influences immune cell function and response to infections. MicroRNA-33 targets the ATP-binding cassette transporter A1 (ABCA1) gene, which is involved in cholesterol efflux [58]. By regulating cholesterol homeostasis, this miRNA enhances immune cell function and improves resistance to bacterial infections [59]. Chronic respiratory diseases caused by *Mycoplasma gallisepticum* have severe consequences for the poultry industry. Zhao et al. [60] identified 45 and 68 DE miRNAs at 3 and 10 days post-infection (dpi) with *Mycoplasma gallisepticum*, respectively, and suggested the mitogen-activated protein kinase pathway as a key regulatory route for the role of miRNAs in regulating disease progression. They also highlighted miR-8, miR-499, and miR-17 families as potentially important in *Mycoplasma gallisepticum* infection. In a follow-up study, Zhao et al. [61] identified miR-99a as a key player in *Mycoplasma gallisepticum* infection through the regulation of NF2-Related Chromatin Remodeling ATPase 5 (SMARCA5). Other important miRNAs for this pathogen include miR-101-3p [62] and miR-19a [63]. MiR-19a regulates the expression of the host Enhancer of Zeste Homolog 2 (EZH2) gene through binding to its 3′ untranslated region [63]. MiR-19a might also suppress the expression of Zinc Finger MYND-Type Containing 11 (ZMYND11) in *Mycoplasma gallisepticum*-infected chicken embryonic lungs and DF-1 cells [63]. MiR-19a can activate the NF-κB signaling pathway and promote the expression of pro-inflammatory cytokines, cell cycle progression, and cell proliferation to defend against *Mycoplasma gallisepticum* infection [63].

##### Regulation of Viral Infections

Viral infections are a major concern in poultry production, and miRNAs have been shown to play a role in the immune response to viral pathogens (Figure 2). Avian influenza is a deadly disease for many bird species with potential effects on human health. Wang et al. (2009) conducted the earliest deep sequencing analysis of miRNA expression in avian influenza virus (AIV)-infected chickens, identifying 73 and 36 DE miRNAs in lung and tracheal tissues, respectively, following low pathogenic H5N3 infection at four days post-inoculation [64]. Several of these DE miRNAs, including miR-146, miR-15, and miR-21, have recognized roles in mammalian immune signaling pathways. Expanding on this foundation, Wang et al. (2012) evaluated host miRNA responses in broiler chickens, proposing miR-34a, miR-122-1, miR-122-2, miR-146a, miR-155, miR-206, miR-1719, miR-1594, miR-1599, and miR-451 as strong regulatory candidates during AIV infection, alongside key genes such as MX Dynamin Like GTPase 1 (MX1), Interleukin 8 (IL-8), Interferon Regulatory Factor 7 (IRF-7), and Tumor Necrosis Factor Receptor Superfamily Member 19 (TNFRS19) [65]. Peng et al. (2015), who analyzed miRNA profiles in chicken embryo fibroblasts challenged with H9N2, reported 48 DE miRNAs, such as miR-146c, miR-181a, miR-181b, miR-30b, miR-30c, miR-30e, and miR-455, that were predicted to target genes involved in the immune response [66]. O’Dowd et al. (2020) identified a total of 224 DE miRNAs (67 upregulated and 157 downregulated) in both cellular and extracellular vesicle fractions derived from chicken tracheal cells subjected to polyI:C stimulation, LPS from E. coli O26:B6, or low pathogenic H4N6 infection [67]. They proposed miR-146a, miR-146b, miR-205a, miR-205b, and miR-449 as promising candidates for development into miRNA-based antiviral therapies or vaccine adjuvants to support control measures against AIV in poultry. It has been reported that miR-26a-5p modulates Melanoma Differentiation-Associated Protein 5 (MDA5) during highly pathogenic AIV infection [68]. A recent study also reported that miR-214 might regulate the Phosphatase and Tensin Homolog (PTEN) in stress-induced immunosuppression, affecting chicken immune response to AIV vaccine [69], while miR-18a-5p can target Neural Precursor Cell Expressed Developmentally Downregulated 9 (NEDD9) to suppress H5N1 AIV [70].

Bursal disease is a highly contagious disease that predominantly affects the bursa of Fabricius in chickens and other birds [71,72]. Infectious bursal disease virus (IBDV) destroys B lymphocytes, attracts T cells, and activates macrophages to target the host immune system [73,74]. Some studies have been devoted to examining the function of miRNAs in this virus infection. Among the first studies, Shen et al. reported that recombinant avian adeno-associated virus (AAAV)-delivered VP1- and VP2-specific miRNAs can efficiently inhibit the replication of IBDV in 8-day-old specific-pathogen-free chicken embryos [75]. Ouyang et al. reported that miR-9 can promote IBDV replication by repressing the production of type I IFN. The authors also reported [76] that miR-2127 functions in IBDV infection via downregulation of Calcium-binding Protein P53 (CHP53) mRNA translation and attenuation of CHP53-mediated antiviral innate immune response against IBDV [77]. Additionally, Fu et al. reported that miR-130b suppresses IBDV replication via directly targeting the viral genome and cellular Suppressor of Cytokine Signaling 5 (*SOCS5*) [78]. Chen et al.(2023) [79] also reported that miR-20b-5p inhibits infectious bursal disease virus replication via targeting Netrin 4. Huang et al. (2024) [80] showed that lncRNA53557 acted as a competing endogenous RNA of miR-3530-5p to relieve the repressive effect of miR-3530-5p on its target genes, such as Signal Transducer and Activator of Transcription 1 (STAT1), MX Dynamin Like GTPase 1 (Mx1), 2′-5′-Oligoadenylate Synthetase-Like (OASL), and ISG15 Ubiquitin Like Modifier (ISG15), to suppress the virus replication. Jiang et al. (2025). [81] also reported that miR-17-5p, miR-20a-5p, and miR-20b-5p are involved in the molecular mechanism of the Programmed Death-Ligand 1 (PD-L1) gene, which is involved in the immune response to IBDV.

Avian leukosis virus (ALV), an Alpharetrovirus of the Retroviridae family, induces tumors like B-cell lymphoma, hemangioma, and myelocytoma in avian hosts [82]. Multiple miRNAs have been reported to be related to different stages of infection and cell types by this virus [59,83,84,85,86]. Li et al. (2012) found seven upregulated miRNAs (miR-221, miR-222, miR-1456, miR-1704, miR-1777, miR-1790, and miR-2127) in livers of ALV-J-infected chickens, potentially tumorigenic, and five downregulated miRNAs (let-7b, let-7i, miR-125b, miR-375, and miR-458) linked to loss of tumor suppression [87]. Li et al. (2014a) confirmed miR-375 downregulation in ALV-J-infected chicken livers, showing its overexpression inhibits DF-1 cell proliferation by targeting Yes1-Associated Transcriptional Regulator (YAP1), Cyclin E1 (CCNE1), and Drosophila Inhibitor of Apoptosis Protein 1 (DIAP1) [85]. Wang et al. [83] identified miR-221, miR-193a, miR-193b, and miR-125b as DE miRNAs in ALV-J liver tumors, with miR-221 and miR-222 promoting tumorigenesis by targeting BCL-2 modifying factor [88]. Li et al. [89] linked upregulated miR-23 in spleens to enhanced ALV-J replication via IRF1 suppression. Li et al. (2017d) reported that miR-34b-5p suppresses MDA5 signaling, promoting ALV-J replication and cell proliferation [90]. Ji et al. (2017a) showed temporal changes in let-7b and let-7i expression in ALV-J-challenged chickens [91]. Zhou et al. (2018) identified 54 DE miRNAs (23 upregulated and 31 downregulated) in CEF cells with ALV-J, REV, or both, with miR-184-3p, miR-146a-3p, miR-146a-5p, miR-3538, and miR-155 involved in virus–vector interaction, oxidative phosphorylation, energy metabolism, and cell growth, based on Gene Ontology (GO) and KEGG pathway analyses [92].

Marek’s disease, caused by Gallid herpesvirus 2, remains a serious threat due to its constantly evolving virulent strains [93]. Various in vitro methods have explored the role of host miRNAs during MDV infection [94,95,96,97,98,99,100,101]. Deep sequencing identified DE miRNAs linked to lymphomagenesis [97,100]. Studies using the oncogenic RB-1B strain observed altered expression of miR-21, miR-26a, miR-103, and miR-219b, which impacted tumor progression and apoptosis [102,103,104,105]. Heidari et al. (2022) revealed 300 novel miRNAs and 54 DE miRNAs associated with immune pathways, such as cytokine signaling, the innate and adaptive immune system, Toll-like receptors, and interleukin pathways [106].

#### 3.1.3. Role of MicroRNAs in Exterior Traits in Chickens

Exterior traits in chickens, such as feather color, comb type, skin pigmentation, and body size, are important for breed identification (https://www.animalgenome.org/cgi-bin/QTLdb/GG/ontrait?class_ID=1, accessed on 20 January 2025), esthetic value, and functional purposes. miRNAs play a critical role in regulating exterior traits in chickens, such as feather color [107,108], comb weight [109], skin pigmentation, body size, and feather development. Specific miRNAs, such as miR-137, miR-675, miR-1, miR-206, miR-143, miR-199a, miR-133, miR-27a, miR-31, and miR-203, have been identified as important regulators of these traits. Understanding the roles of these miRNAs provides valuable insights into the molecular mechanisms underlying exterior traits and offers potential applications for improving breed characteristics in poultry breeding programs.

##### Feather Color

Feather color is one of the most noticeable exterior traits in chickens and is determined by the type and distribution of pigments, such as melanin. miRNAs have been shown to regulate genes involved in melanogenesis, the process of melanin production [110,111]. Recent studies have suggested miRNAs are important regulators of feather development in chickens and are likely involved in feather color by modulating genes in pigmentation pathways, although direct evidence for specific miRNAs controlling feather color remains limited. For example, miRNAs have been shown to regulate feather morphogenesis and epidermal differentiation in chickens [108]. Studies have identified 19 miRNA–mRNA pairs involved in feather and scale development, targeting genes related to cell signaling (e.g., ALDH1A3), cell adhesion (e.g., TNC), and structural proteins like β-keratins, which are important components of feathers [108]. Fang et al. observed differential expression of miRNAs during feather development when comparing early- and late-feathering chickens [107]. The authors identified 31 miRNAs with significant differences and suggested some interaction pairs, such as miR-1574-5p/NR2F, miR-365-5p/JAK3, and miR-365-5p/CDK6, might play important roles in hair or feather formation.

##### Comb Type

miRNAs have been implicated in various aspects of chicken biology, but direct studies linking specific miRNAs to comb development are limited. However, recent research has identified at least one miRNA, miR-6633, as a candidate associated with comb weight in chickens. This was discovered through whole-genome resequencing and quantitative trait loci mapping, which found that regions containing miR-6633 are associated with variation in comb weight [109].

##### Skin Pigmentation

Skin pigmentation is another important exterior trait in chickens, particularly in breeds with black skin, such as Silkie chickens. miRNAs have been shown to regulate genes involved in skin pigmentation [112]. In other species, miR-141-3p and miR-200a-3p were reported to directly target microphthalmia-associated transcription factor (MITF) [112]. Overexpression of these miRNAs suppresses melanogenesis and tyrosinase activity, while their downregulation is associated with increased melanin synthesis [112]. Another miRNA, miR-206, regulates pigmentation by targeting the Mc1r gene, which is upstream of MITF [113] in Koi carp. Lower miR-206 leads to higher Mc1r and increased melanin synthesis [113]; in this species, the role of miR-206 in chicken pigmentation remains to be validated.

##### Body Size

Body size is an important exterior trait in chickens, particularly in meat-type breeds. miRNAs have been shown to regulate genes involved in growth and development. MicroRNA-24-3p, novel miR-133, miR-16, miR-223, miR-146b-3p, miR-205a, miR-206, let-7b, and miR-21 are among the most significant miRNAs implicated in the regulation of body size in chickens. They exert their effects by modulating muscle cell proliferation/differentiation and influencing key growth hormone pathways. For example, miR-146b-3p targets the growth hormone receptor (GHR), directly influencing growth signaling [114]. Changes in miR-16 expression have been linked to differences in muscle mass and growth rates, possibly through regulation of signaling pathways such as MAPK, PI3K, Akt, Wnt, and insulin signaling [115].

#### 3.1.4. Role of MicroRNAs in Physiological Traits in Chickens

MicroRNAs play a significant role in various physiological processes in chickens, including metabolism, stress response, thermoregulation, and immune function. These physiological traits are crucial for maintaining health, productivity, and adaptability in poultry.

##### Metabolism

Metabolism is a fundamental physiological process that influences growth, energy balance, and nutrient utilization in chickens. miRNAs are central regulators of metabolism in chickens, influencing processes such as lipid metabolism, adipogenesis, hepatic metabolic transitions, and energy homeostasis across tissues like liver, muscle, and adipose tissue. miRNAs regulate nearly all aspects of chicken metabolism, including the switch from embryonic lipid-based metabolism to post-hatch carbohydrate-based metabolism [116]. For instance, miR-33 is directly involved in hepatic lipogenesis in chickens, as it regulates fatty acid synthase (FASN) and represses the fat mass and obesity-associated gene, influencing lipid metabolism and storage during development [116]. The miR-216/miR-217 cluster is upregulated in the livers of chickens with fatty liver syndrome (FLS) and in hepatocytes exposed to free fatty acids. Overexpression of miR-216a/b and miR-217-5p leads to increased lipid accumulation in liver cells. These miRNAs directly target genes such as HACD2, FBXO8, and TM9SF3, suppressing their expression. This suppression activates the PPAR/SREBP signaling pathway, a central regulator of lipid metabolism, promoting lipogenesis and fat storage in the liver [117]. MicroRNA-130b-3p and miR-218-5p regulate lipid metabolism by targeting INSIG1 and INSIG2, which are key components of the INSIG-SCAP-SREBP pathway. This pathway is essential for controlling triglyceride and cholesterol biosynthesis in the liver [118]. MicroRNA-375 is reported to target the recombination signal binding protein for immunoglobulin kappa J region (*RBPJ*) gene to upregulate lipid metabolism and inhibit cell proliferation involved in chicken fatty liver formation and inheritance [119].

##### MicroRNAs and Stress Response

Stress response is a critical physiological trait that influences the health and productivity of chickens. miRNAs have been shown to regulate genes involved in stress response. miRNAs play a crucial role in the chicken stress response by regulating gene expression in response to various stressors, including heat, cold, immune challenges, and nutritional deprivation. Thermoregulation is an important physiological trait that influences the adaptability and productivity of chickens, particularly in extreme environmental conditions. miRNAs play a critical role in regulating heat stress responses and resilience traits in chickens by controlling key processes such as heat shock response, oxidative stress, apoptosis, thermoregulation, and immune function. MicroRNA-34a and miR-449c are differentially expressed in the spleen under both heat and cold stress. These miRNAs target immune-related genes, such as interleukin 2 (IL-2) and interleukin 12α (IL-12α), directly influencing cytokine-mediated immune responses during stress. Some other miRNAs related to heat stress, such as miR-22, miR-23a, miR-27a, miR-30a-5p, miR-92a, miR-146a, and miR-155, were upregulated in heat stress, while miR-let-7f and miR-181a were downregulated [120]. These changes are associated with the modulation of heat shock proteins, antioxidant defenses, and immune signaling pathways. Feed deprivation and other management-related stressors rapidly alter the plasma miRNome, with stress-responsive miRNAs like miR-204, miR-let-7f-5p, and miR-122-5p showing changes that reflect metabolic adaptation and genetic background [121].

### 3.2. MicroRNAs in Other Poultry Species

#### 3.2.1. MicroRNAs in Turkey

The turkey genome was first sequenced in 2010 [122], marking a significant milestone in avian genetics. This progress provided a foundation for studying key biological and economic traits, contributing to improvements in turkey health, productivity, and disease resistance. Although research on miRNAs in turkeys remains limited compared to other poultry species like chickens, existing studies offer a promising foundation for understanding their potential functions in various biological processes. One notable aspect is the impact of stress on the immune system, where certain miRNAs have been identified as regulators of stress responses. For example, miR-22, miR-155, and miR-365 have been used as biomarkers to assess stress expression levels following transportation [123]. Among these, miR-155 is particularly recognized for its role in modulating immune responses [123]. Additionally, miRNAs play a role in turkey muscle development. Velleman and Harding (2017) demonstrated that miR-128 and miR-24 regulate the proliferation and differentiation of myogenic satellite cells [124]. These satellite cells migrate and align to form new myofibers or contribute nuclei to existing myofibers, supporting muscle growth in turkeys [124]. Future research should aim to thoroughly investigate the functions of miRNAs in turkeys. Such efforts hold immense potential for applications in enhancing the health and productivity of this species.

#### 3.2.2. MicroRNAs in Duck

Meat quality, stress resistance, and egg productivity are important aspects of duck farming, and miRNA research helps determine the genetic influence on these traits. For skeletal muscle development, miRNA-1 promotes myoblast differentiation, while miRNA-133 maintains cells in an undifferentiated state and supports cell growth [125]. In stress response, 41 conserved miRNAs and three novel miRNAs exhibit differential expression in duck intestines following transportation, indicating their involvement in regulating stress adaptation [126]. In addition, miR-31-5p and novel-273 show potential as biomarkers for evaluating sperm quality [127]. Moreover, in lipid metabolism, N-miR-16020 and miR-144 regulate the expression of key genes, including FASN and Elongation of Very Long Chain Fatty Acids Protein 6 (ELOVL6), by binding to their 3′-untranslated regions, which contain regulatory elements affecting mRNA stability and translation [128]. Wang et al. also reported the importance of the miRNA networks in abdominal fat deposition in ducks [129]. Advanced techniques like m6A and miRNA analysis uncover how elements jointly regulate embryonic muscle development [130], while imaging tools such as transmission electron microscopy (TEM) and nanoparticle tracking analysis (NTA)helped characterize small extracellular vesicles SPEVs [127]. These approaches highlight the critical role of miRNAs in regulating diverse biological processes in ducks.

#### 3.2.3. MicroRNAs in Geese

In 2015, the research conducted by Lu and colleagues on the goose genome established a robust foundation, which has led to subsequent studies on ducks and other waterfowl. This research also opened miRNA studies on geese, addressing critical production-related aspects such as fatty liver disease, feather follicle development and quality, and egg production [131]. In ovarian follicle development, specific miRNAs are differentially expressed, targeting genes associated with cellular processes, metabolism, and molecular binding [132]. For feather follicle development, miR-144-y may significantly influence feather pigmentation, as it has been associated with skin and feather follicle development in Zhedong white geese, indicating its potential involvement in feather growth [133]. In muscle development, miR-16-1 regulates the Transmembrane Protein 8C (Tmem8C) gene, with negative correlations observed between Tmem8C expression and specific miRNAs in both breast and leg muscles [134]. Additionally, miRNAs such as miR-203a and miR-125b-5p play a role in lipid metabolism regulation in the liver, shedding light on mechanisms underlying fatty liver disease [135]. These discoveries have been enabled by modern genomic technologies applied to geese. Network analyses further deepen the scope and accuracy of these studies, laying the groundwork for understanding miRNA roles in geese [136,137].

#### 3.2.4. MicroRNAs in Quails

MicroRNAs play a crucial role in quail development, particularly in embryogenesis and muscle formation. During early embryonic stages, miRNAs such as let-7a, miR-122, and miR-125b show differential expression, indicating their involvement in key developmental processes [138]. MiRNA-122, highly expressed in the developing and mature liver, regulates metabolic functions like lipid metabolism. However, studies have shown a significant decrease in miR-122 expression between 19 and 30 h of incubation. Similarly, miR-125b expression declines during this period, suggesting its downregulation contributes to somite formation, a critical step in embryonic development [138]. In muscle development, miRNAs such as miR-1, miR-133, and miR-206 are essential for differentiation, muscle growth, and apoptosis regulation, with their expression varying across developmental stages [139]. While research on miRNAs in quail is less developed compared to other avian species, advances in genomic technologies hold great potential to uncover deeper regulatory mechanisms.

## 4. Prospects and Challenges

### 4.1. Prospects

The study of miRNAs in poultry holds immense potential for advancing various aspects of poultry science and industry. Several promising applications and prospects emerge from ongoing research in this field. Incorporating miRNA profiles into genomic selection programs can significantly enhance the precision and efficiency of breeding strategies. By identifying miRNAs associated with desirable traits such as growth rate, feed efficiency, and disease resistance, breeders can make more informed decisions. This approach can lead to the development of superior poultry lines with optimized performance and health characteristics. The targeted delivery of miRNA mimics or inhibitors presents a novel therapeutic approach for managing diseases and improving growth and reproduction in poultry. MicroRNA mimics can restore the function of downregulated miRNAs, while inhibitors can suppress the activity of overexpressed miRNAs. These interventions can be tailored to address specific health issues, such as viral infections, metabolic disorders, and reproductive challenges, thereby enhancing overall poultry welfare and productivity. Understanding the interactions between miRNAs and epigenetic factors, such as DNA methylation and histone modifications, can open new avenues for trait improvement. MicroRNAs can influence epigenetic landscapes by targeting enzymes involved in these modifications, thereby regulating gene expression patterns. Exploring these interactions can lead to innovative strategies for modulating epigenetic states to achieve desired phenotypic outcomes, such as improved growth rates, enhanced immune responses, and better stress resilience. miRNAs can also be leveraged to optimize nutritional strategies in poultry. By understanding how miRNAs respond to different dietary components, researchers can develop feed formulations that modulate miRNA expression to promote growth, enhance nutrient utilization, and improve gut health. This approach can contribute to more sustainable and efficient poultry production systems.

Genome editing technologies like CRISPR/Cas9 are transforming miRNA-based poultry breeding by enabling precise modifications to miRNA genes and their regulatory regions [140,141]. These edits can enhance traits such as disease resistance, growth efficiency, and immune function [142]. For instance, genome editing has created chicken resistance to AIV via editing of the ANP32 gene family [143]. The edited chickens exhibited markedly reduced viral replication and transmission following exposure to the H9N2 strain, with resistance observed even under high viral challenge conditions. Importantly, genetic modifications were introduced into germline cells, allowing for stable inheritance of the resistance trait across generations. This strategy presents a promising alternative to conventional vaccination, particularly in the context of rapidly mutating influenza viruses and biosecurity challenges in poultry production systems. Given the regulatory role of miRNAs in host–pathogen interactions and immune modulation, genome editing of specific miRNA loci may offer an additional avenue for enhancing antiviral resistance in poultry. Machine learning and deep learning, as vital parts of AI, have shown great potential for miRNA discovery and target prediction [144,145]. AI might revolutionize poultry breeding and health management by enabling the identification and prediction of key regulatory molecules that influence disease resistance and productivity [146,147]. Machine learning models can analyze RNA-seq data to uncover genes linked to stress response in chickens [148]. The integration of AI with genome editing further accelerates miRNA-based advancements in poultry health and breeding. AI can guide CRISPR design by predicting off-target effects and optimizing guide RNAs. Machine learning models can analyze genomic and phenotypic data to identify birds with beneficial miRNA edits, streamlining selection for traits like disease resistance and egg production. Moreover, AI systems can monitor edited flocks in real time, using miRNA expression data to detect stress or disease early and ensure the long-term success of genetic interventions. This synergy between AI and genome editing paves the way for more resilient, productive poultry populations.

### 4.2. Challenges

**Figure 3:**
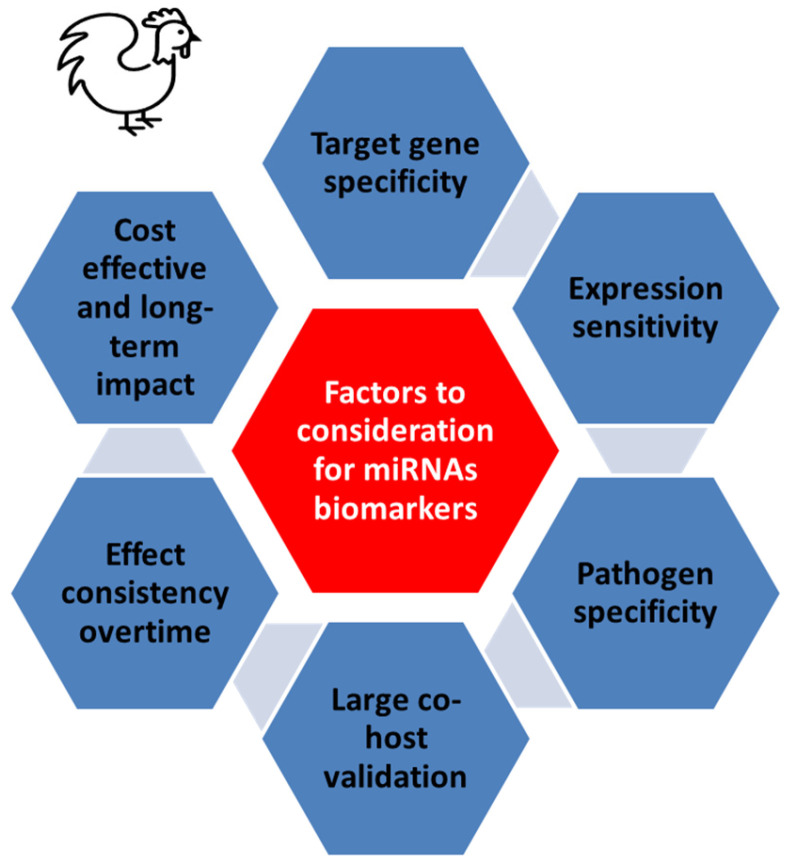
Major factors to consider in developing miRNAs as biomarkers in poultry. Factors include target gene and pathogen specificity, expression sensitivity, validation across hosts, consistency over time, and cost-effectiveness.

Changes in miRNA expression profiles can indicate the presence of infections, metabolic imbalances, or other health issues. Developing miRNA-based diagnostic tools can enable timely interventions, reducing the impact of diseases on poultry flocks and improving overall flock health management. However, several challenges must be considered for cost-effective methods to develop miRNAs as biomarkers in poultry (Figure 3). Factors include target gene and pathogen specificity, expression sensitivity, validation across hosts, consistency over time, and cost-effectiveness. Additionally, high development and production costs are a major barrier, as identifying relevant miRNAs, validating their efficacy, and manufacturing stable, high-purity molecules require significant investment [149]. Unlike human medicine, where expensive treatments may be justified for individual patients, poultry farming demands affordable solutions for entire flocks. Additionally, extensive field trials are needed to validate these technologies across diverse breeds and environments, further increasing costs and complicating their commercial viability.

Although significantly improved recently, the delivery system limitations also hinder the adoption of miRNA-based interventions [149,150]. MicroRNAs are inherently unstable in vivo and struggle to penetrate cell membranes, making targeted delivery to poultry tissues—such as respiratory or immune cells—particularly difficult. While human medicine has explored lipid nanoparticles and viral vectors [149], these methods are not yet scalable or cost-effective for poultry. Common administration routes like feed or water are unsuitable due to degradation risks, and injectable methods are impractical for large-scale operations. Moreover, miRNAs’ ability to regulate multiple genes raises concerns about off-target effects, such as unintended impacts on growth or reproduction, which are critical traits in poultry farming.

Regulatory and poultry-specific challenges further complicate implementation. Approval processes for veterinary therapeutics are rigorous, requiring extensive safety and environmental testing [151]. Concerns about residues in meat or eggs and potential environmental shedding add layers of scrutiny, especially amid public skepticism toward nucleic acid-based technologies [152]. The lack of precedent for miRNA use in food animals means regulatory pathways are unclear, delaying progress. Additionally, miRNAs’ context-dependent roles across breeds and environments make it difficult to design universally effective solutions. To overcome these hurdles, future efforts must focus on developing poultry-optimized delivery systems, refining regulatory frameworks, and fostering collaboration among researchers, industry, and policymakers.

## 5. Conclusions

In conclusion, the integration of miRNA research into poultry science offers exciting opportunities for advancing breeding, health management, and productivity. Continued exploration of miRNA functions and applications will undoubtedly contribute to the development of innovative solutions that benefit the poultry industry and enhance food security.

## Figures and Tables

**Table 1:** Genome and miRNAs in different poultry species.

Name	Scientific Name	Genome Size (Gb)	Year of Complete Genomics	Number of Genes	Number of miRNAs	Version of Genome Assembly in NCBI
Chicken	*Gallus gallus*	1.1	2004	18,023	474	GCF_016699485.2
Turkey	*Meleagris gallopavo*	1.1	2010	17,974	353	GCF_000146605.3
Duck	*Anas platyrhynchos*	1.3	2021	17,676	~300	GCF_047663525.1
Goose	*Anser anser*	1.3	2014	~19,000	~250	GCA_964211835.1
Quail	*Coturnix japonica*	1.24	2013	~17,000	~200	GCF_001577835.2
Guinea Fowl	*Numida meleagris*	1	2021	21,846	~200	GCF_002078875.1
Ostrich	*Struthio camelus*	1.5	2015	23,381	Data limited	GCA_040807025.1
Emu	*Dromaius novaehollandiae*	1.5	2019	24,513	Data limited	GCF_036370855.1
Pheasant	*Phasianus colchicus*	1	Genome incomplete	19,413	Data limited	GCF_004143745.1
Peacock	*Pavo cristatus*	1	Genome incomplete	15,970–17,490	540	GCA_045791835.1

**Table 2:** MicroRNAs related to production traits in chicken.

Trait	miRNAs	Target Genes
Muscle growth	miR-1	*HDAC4*
	miR-206	*Pax7*
	miR-133	*SRF*
	miR-27a	*PPARγ*
	miR-499	*SOX6*
	miR-208	*MSTN*
	miR-29	*IGF-1*
	miR-486	*PTEN*
Egg production and quality	miR-202-5p	*BMPR2*
	miR-26a	*PTEN*
	miR-181a	*ZP3*
	miR-143	*ERK5*
	miR-21	*PDCD4*
	miR-155	*SOCS1*
	miR-146a	*IRAK1*
Feed efficiency	miR-15a	*FOXO1, PDPK1,*
		*PRKAR2A*
	miR-142-5p	*FOXO3*

**Table 3:** MiRNAs related to the immune system and health in chicken.

Disease	Pathogens	Tissues	MiRNAs
Marek’s disease	Gallid herpesvirus 2	Spleen and liver	miR-221, miR-140, miR-199, miR-181a, miR-146b, miR-146c, and miR-26a
		Spleen	miR-15, miR-456 and let-7i
		Spleen	miR-21
		Spleen and liver	miR-21
		Spleen	miR-26a
		Spleen and liver	miR-103
		Spleen and liver	miR-219b
Avian leukosis virus	Avian leukosis virus	Liver	(miR-221, miR-222, miR-1456, miR-1704, miR-1777, miR-1790, and miR-2127), let-7b, let-7i, miR-125b, miR-375, and miR-458
		Liver	miR-375
		Liver	ga-miR-221, miR-193a, miR-193b, and miR-125b
		Liver	miR-221, miR-222,
		Liver	miR-23
		Liver	miR-34b-5p
		Liver	let-7b and let-7i
Bursal disease	Bursal disease virus		miR-9
			miR-2127
			miR-130b
Avian influenza	Avian influenza viruses	Lung and trachea	as miR-146, miR-15 or miR-21
		Lung	-miR-34a, 122–1, 122–2, 146a, 155, 206, 1719, 1594, 1599, and 451,
		Embryo fibroblasts	miR-146c, miR-181a, miR-181b, miR-30b, miR-30c, miR-30e, and miR-455, miR-1599 and miR-1416
Chronic respiratory disease (CRD)	Mycoplasma gallisepticum		miR-8 family, miR-499 family, miR-17 family
			miR-99a
			miR-101-3p
		Chicken embryonic lungs and DF-1 cells	miR-19a

## Data Availability

No new data were created or analyzed in this study. Data sharing is not applicable to this article.
